# Structural and electrochemical insights into bismuth-based metal organic framework for capacitive applications

**DOI:** 10.1038/s41598-025-23923-x

**Published:** 2025-12-11

**Authors:** Mona Elfiky, Aya Elleboudy, Nehal Salahuddin

**Affiliations:** Department of Chemistry, Faculty of science, Tanta University, Tanta, Egypt

**Keywords:** Sustainable energy storage, Bi-1,4-benzenedicarboxylate nanosheets, Metal-organic frameworks electrodes, Solvothermal synthesis., Chemistry, Energy science and technology, Materials science

## Abstract

The growing need for efficient energy storage has revealed key limitations in conventional battery-type electrodes, particularly their low electrical conductivity and limited cycling stability. To address this issue, a Bi-1,4-benzenedicarboxylate (Bi-OF) metal organic framework was synthesized in the form of nanosheets using a simple solvothermal method. The structure of Bi-OF was examined using Fourier transform infrared spectroscopy (FTIR), X-ray diffraction (XRD), scanning electron microscopy (SEM), and transmission electron microscopy (TEM). The Bi-OF material was used to modify the glassy carbon electrode (GCE), and its electrochemical performance was systematically evaluated. Its electrochemical behavior was evaluated through cyclic voltammetry (CV), charge-discharge (CD) testing, and electrochemical impedance spectroscopy (EIS). The Bi-OF electrode achieved a high specific capacitance of 1797.88 C·g⁻¹ (1284.2 F·g⁻¹) at 2.0 A·g^−1^. It also showed low internal resistance (110 Ω) and maintained 84.7% of its initial capacitance after 3000 cycles. These results suggest that Bi-OF is a promising candidate for high-performance and environmentally friendly supercapacitor applications.

## Introduction

The growing use of portable devices, electric appliances, and electric transportation will significantly boost global electricity demand^1^. Much of this energy currently comes from fossil fuels, leading to increswd pollution and greenhouse gas emissions^2^. Therefore, there is an urgent need to rapidly develop solar energy, geothermal energy, and wind power to meet this rising demand^3^. The widespread adoption of solar and wind power depends on advancements in fuel cells, electrochemical cells, and battery technologies due to their intermittent nature. Energy storage technologies play a crucial role in daily life, powering portable electronics, hybrid vehicles, and cell phones^4,5^. Their performance, assessed through power density (P) and energy density (E) in Ragone plots^6^^,^^7^, varies widely: batteries offer high P and E but require longer charging times, while conventional capacitors have lower E and P but faster charge-discharge cycles^8^. Supercapacitors, blending characteristics of both batteries and capacitors, provide a balanced solution with sufficient E and P, meeting diverse electronic device requirements^9^, making them crucial for sustainable energy systems^10^.

Supercapacitors are categorized into two main types based on their charge storage mechanisms: electrical double-layer capacitors (EDLCs) and pseudocapacitors. EDLCs offer high power density and stability but have lower energy densities due to the limited capacitance of carbon-based electrodes^11^. Pseudocapacitors, on the other hand, achieve higher energy densities through fast, reversible redox reactions. Emerging research focuses on a third mechanism known as the battery-like mechanism, combining aspects of batteries and supercapacitors to bridge their capabilities^12^. The battery-like mechanism, or Faradaic mechanism, involves storing charge through redox reactions at electrode surfaces, unlike EDLCs, which store charge electrostatically. This allows for higher energy densities by utilizing reversible chemical reactions. Battery-like supercapacitors, with rapid charge and discharge cycles, are promising for high power and energy storage applications such as electric vehicles and grid energy storage^13^. They combine the high-power density and fast charge/discharge of supercapacitors with the high energy density of batteries. Research is focused on improving electrode material stability and ion transport to enhance their potential for sustainable energy storage. Recently, conventional battery-type electrode materials such as transition-metal oxides (sulfides^14^, nitrides^15^ and conducting polymers^16^, have been utilized but fall short of meeting these requirements due to their inadequate electronic conductivity, low specific capacitance, and limited cycling stability. Therefore, there is a pressing need to develop new materials for high-performance supercapacitors.

Metal-organic frameworks (MOFs) have garnered growing interest as potential electrode materials for supercapacitors due to their large specific surface areas, adjustable pore structures, and versatile architectures^17^. These characteristics facilitate thorough interaction with the electrolyte and rapid ion diffusion, which are crucial for sustainable energy storage applications, including gas separation, catalysis, chemical sensors, and heat storage^18–22^. Hence, MOFs can be directly employed as electrode materials for supercapacitors. Furthermore, the preparation of MOFs is facilitated using inexpensive Fe, Co, and Ni salts as starting materials that contain potential pseudocapacitive redox sites^12,17^. Notably, the process of calcining MOFs to produce metal-based electrode materials unavoidably alters the framework of the MOFs to some extent, leading to a reduction in specific surface area and active redox sites. Consequently, utilizing MOFs directly as supercapacitor electrodes allows for the full utilization of their advantages. The organic linkers used in MOFs, such as benzene-1,3,5-tricarboxylic acid (H_3_BTC) or 1,4-benzenedicarboxylate, may pose environmental hazards depending on their chemical nature and stability. These organic compounds can be toxic and may degrade into harmful byproducts under certain environmental conditions. Proper handling and disposal of these chemicals are crucial for minimizing environmental contamination. Bismuth, belonging to the VA group of the periodic table, possesses a valence electronic configuration of 6s^2^ 6p^3^, providing a rich array of valence states conducive to high capacity. Bismuth is relatively abundant in the earth’s crust compared to other heavy metals, which makes it more accessible for large-scale extraction and use. It is typically obtained as a secondary byproduct of other metal refining processes, such as lead and copper extraction, which positions it favorably for recycling ^23^. This secondary sourcing reduces the environmental impact associated with primary mining activities and lowers the overall energy consumption required for its production. Efficient recycling processes can recover bismuth from scrap materials, manufacturing residues, and end-of-life products, thereby closing the loop on its lifecycle and reducing waste. Moreover, as one of the less toxic heavy metals, bismuth is often recognized for its environmental and health safety compared to other heavy metals such as lead, cadmium, or mercury. However, bismuth compounds can still pose risks, particularly if they are not managed properly during synthesis, usage, and disposal. The environmental impact of bismuth extraction and processing should also be considered, as mining activities can lead to habitat destruction and pollution if not conducted responsibly. On the other hand, Bismuth is more affordable compared to many other heavy metals, partly due to its status as a byproduct in mining operations. This lowers the initial cost of raw materials, contributing to the overall economic feasibility. Recent studies found specific capacitances for different metal 1,4-benzenedicarboxylate frameworks: Mn-1,4-benzenedicarboxylate at 64.5 F. g^−1^ (0.25 A/g)^24^, vanadium V-1,4-benzenedicarboxylate at 572.1 F. g^−1^ (0.5 A/g)^25^, and Co-1,4-benzenedicarboxylate up to 1159 F. g^−1^ (0.5 A/g)^26^.

Bismuth-based materials exhibit a large theoretical specific capacity and can function across a wide potential range in alkaline environments^27^. Wu et al.^28^, Xu et al.^29^, Yu et al.^30^, Qin et al.^31^, El-Sabban et al.^32^ have sucessfully synthesized various Bi-based materials, such as Bi-Bi_2_O_3_/CNT core-shell structures, 3D activated carbon fiber paper/α-Bi_2_O_3_, carbon-coated Bi_2_O_3_ microrods, Bi_2_Se_3_@C, and Bi_2_S_3_/sulfur-doped g-C_3_N_4_, exhibiting specific capacitances of 850 F. g^−1^ (1 A. g^−1^), 906 C. g^−1^ (1 A. g^−1^), 1378 C. g^−1^ (0.5 A. g^−1^), 565.9 C. g^−1^ (1 A. g^−1^), and 670 F. g^−1^ (1 A. g^−1^), respectively. The actual conductivity of MOFs used in supercapacitors depends on factors such as complex concentration, specificity of the basic medium, and temperatur. Recently, Wang et al.^33^ successfully prepared Bi-OF materials with a 2:1 molar ratio of Bi^3+^ to benzene-1,3,5-tricarboxylic acid (H_3_BTC), demonstrating a specific capacity of 896.1 C. g^−1^ (0.5 A. g^−1^). Furthermore, the Bi-OF electrode maintained 71.2% capacity retention after 2000 cycles at 2.0 A. g^−1^. Linear metal-organic frameworks (MOFs) offer advantages due to their elongated and interconnected one-dimensional channels or pores, facilitating efficient diffusion of ions and enhancing framework stability under various conditions^16^. The ligand-to-metal ratio significantly impacts electronic communication efficiency on MOF surfaces. As reported by Wang et al^33^., a 2:1 ratio (H_3_BTC : Bi^+3^) in Bi-OF could enhance metal ion connectivity but might limit charge delocalization compared to a 1:1 ratio. Conversely, a 1:1 ratio (1,4-benzenedicarboxylate: metal) typically forms coordination polymers with well-defined geometries and efficient electronic communication between metal centers, promoting conductivity. Recent studies have highlighted the superiority of metal-1,4-benzenedicarboxylate complexes over metal-benzene-1,3,5-tricarboxylic acid complexes in supercapacitors, demonstrating higher specific capacitance, charge storage capacity, faster charge-discharge kinetics, cycling stability, and enhanced density of active sites for charge storage. These unique structural characteristics contribute to superior electrochemical performance and charge storage capabilities^34,35^.

Herein, porous Bi-OF nanosheets were synthesized via a simple solvothermal process with a Bi^3+^/H_3_BDC molar ratio of 1:1, yielding porous nanosheets with outstanding electrochemical performance. Unlike conventional MOF-derived electrodes that require calcination often, leading to structural degradation, the Bi-OF material was directly applied to modify a glassy carbon electrode (GCE). Electrochemical evaluation revealed a high specific capacitance of 1797.88 C·g⁻¹ (1284.2 F·g⁻¹) at 2.0 A. g^−1^ and good cycling stability, retaining 84.7% capacity after 3000 cycles at 5.0 A. g^−1^. This calcination-free approach enhances redox site accessibility and preserves structural integrity, offering a superior alternative to traditional MOF-based electrode fabrication.

## Experimental section

### Materials and methods

High-quality chemicals were procured in their pure state and utilized without further purification: 1,4-benzenedicarboxylic acid (BDC, 99.0%), N, N-dimethyl formamide (DMF, ≥ 99.8%), glacial acetic acid (CH_3_COOH, Ac, 98.0%), polymeric perfluorosulfonic acid (nafion, NF, 5.0%), potassium hydroxide (KOH, 98.0%), and isopropyl alcohol (ISA, 99.5%).

Fourier transform infrared spectroscopy (FTIR) analysis was performed using a Shimatzu FTIR-8101 A instrument in the range of 4000–400 cm^−1^ to assess transmittance, while XRD measurements were conducted with a Philips PW 1710 instrument equipped with Cu-Kα radiation (λ = 1.54060 Å) and operated at a voltage of 40 kV to determine the crystallinity of Bi-OF. Scanning electron microscopy (SEM) and high-resolution transmission electron microscopy (HR-TEM) were employed to examine the morphology of Bi-OF powder using an SU8000 instrument, and JEM-2100 JEOL instrument, respectively. Additionally, the prepared Bi-OF powder underwent degassing at 150 °C for 2 h under vacuum before determining the specific surface area using the Brunauer-Emmett-Teller (BET) method. Moreover, cyclic voltammetry (CV), electrochemical impedance spectroscopy (EIS), and charge-discharge measurements were conducted using a computer-controlled potentiostat/galvanostatic model CS3104 (China) in the microanalysis unit at the Faculty of Science, Tanta University to study the supercapacitance performance.

### Preparation of Bi-1,4-benzenedicarboxylate nanosheets framework (Bi-OF)

The solvothermal method was employed to fabricate Bi-1,4-benzene dicarboxylate (Bi-OF). Specifically, a solution containing 1 mmol of Bi(NO_3_)_3_·5H_2_O and 1 mmol of BDC was prepared in a mixture of solvents (DMF: double deionized water (DDW): AC) with a molar ratio of (5:1:3), followed by continuous stirring for half an hour. Subsequently, the reaction mixture was inserted in autoclave and allowed to proceed in an oven at 120 °C for 24 h. The resulting grey powder (Bi-OF) was collected via centrifugation, washed with DDW, and then dried in an oven at 70 °C for 24 h.

### Electrochemical measurements

A glassy carbon electrode (GCE) of 3.0 mm diameter underwent polishing with 0.05 μm alumina powder to achieve a highly reflective surface, which was thoroughly rinsed before utilization. Subsequently, 1 mg of Bi-OF was dispersed in a mixture of [1.0 NF: 1.0 ISA mL], and 5µL of this dispersed mixture was added onto the surface of clean GCE, followed by drying at 60 °C for 2 h. Electrochemical measurements were carried out in a 10.0 mL solution of 1.0 M KOH using a three-electrode system comprising Hg/Hg_2_Cl_2_ and platinum electrodes. Cyclic voltammetry (CV) measurements were performed within the voltage range of −1.50 V to 0.10 V at a scan rate of 50 mV. s^−1^, and galvanostatic charging/discharging (GCD), while electrochemical impedance spectroscopy (EIS) measurements were conducted across a frequency range of 0.1 to 10^6^ Hz. Moreover, the specific capacitance (SCs) values of the modified GCE were determined using the Eqs. (1) and (2).

1 $$\:{C}_{s}=\frac{area\:under\:curve}{m\:\varDelta\:V\:v}$$

2 $$\:\:\:\:\:\:\:{C}_{s}=\frac{I\:\varDelta\:T}{\varDelta\:V}$$

The scan rate, represented as *v* (mV. s^−1^). ΔV (V) stands for the range of applied potential, m (mg) denotes the mass of the sample on the surface of sensor, I (A) indicate the current charge-discharge value, and ΔT(s) represents the time taken for charge-discharge.

## Results and discussion

### Characterizations

**Fig. 1:**
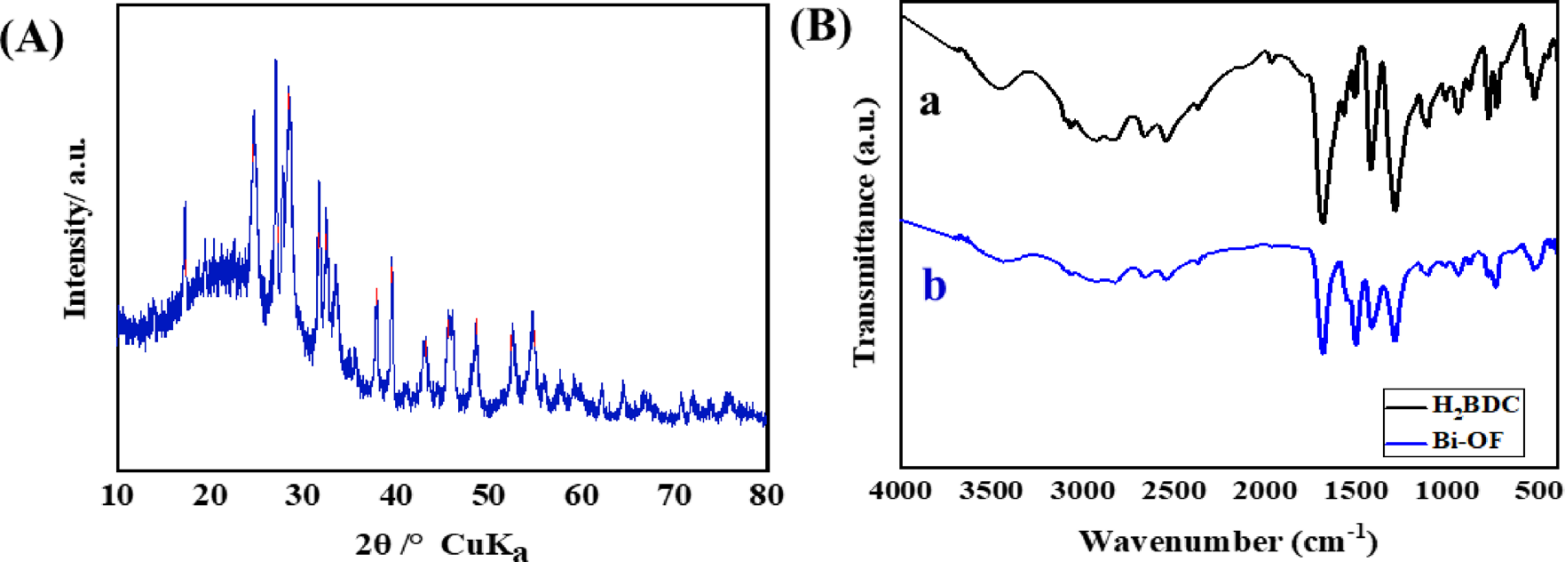
(**A**) XRD pattern of Bi-OF powder. (**B**) FT-IR spectra of (a) H_2_BDC ligand, and (b) Bi-OF powder.

XRD pattern of the synthesized Bi-OF (Fig. 1A) confirms its high crystallinity and phase purity. The observed diffraction peaks correspond to the rhombohedral crystal structure of bismuth, assigned to space group R-3 m (ICDD Card No. 01–085-1329). Prominent reflections at 2θ values of 22.5⁰, 23.8⁰, 27.16⁰, 37.95⁰, 39.61⁰, 48.70⁰, and 64.51⁰ are indexed to the (003), (101), (012), (104), (110), (202), and (122) planes, respectively. Among these, the (012) plane is identified as the most exposed facet. The nanosheets exhibit a crystalline nature, with interplanar spacings of 0.22 nm and 0.32 nm corresponding to the (110) and (012) planes, respectively. Based on the Scherrer equation applied to the (012) peak at 2θ = 27.16°, the average crystallite size was estimated to be approximately 16.5 nm. Additionally, lattice parameters were calculated using Bragg’s law and the rhombohedral interplanar spacing equation, yielding a d-spacing of 0.328 nm for the (012) plane^36^. These results support the formation of well-defined Bi-OF nanosheets.

As illustrated in **(**Fig. 1B_a_), specific absorption bands characteristic of the H_2_BDC ligand were identified. Notably, strong absorption bands at approximately 1503 cm^−1^ and 1416 cm^−1^ were assigned to the asymmetric and symmetric stretching vibrations (ν_COO−_) of the benzenedicarboxylate anion, respectively. As demonstrated in (Fig. 1B_b_), these bands exhibited shifts from 1503 cm^−1^ to 1497 cm^−1^ and from 1422 cm^−1^ to 1412 cm^−1^, which may indicate the deprotonation of the carboxylate groups of the benzenedicarboxylate anion, followed by coordination with Bi cations. Furthermore, the observed shift in the out-of-plane ring vibration within the 1,4-substituted bond core of the 1,4-benzenedicarboxylate molecules from 728 cm^−1^ to 738 cm^−1^ suggests effective coordination of Bi cations with the 1,4-benzenedicarboxylate (1,4-BDC). Interestingly, the observed split in the strong absorption band within the 3500–3300 cm^−1^ range was attributed to the vibrational modes of coordinated water molecules (OH). As depicted in (Fig. 1B_b_), the shift of the –OH bands from 3458 cm^−1^ to 3439 cm^−1^ further supports the notion of strong coordination between the COO^−^ groups of the ligand and Bi^3+^.

**Fig. 2:**
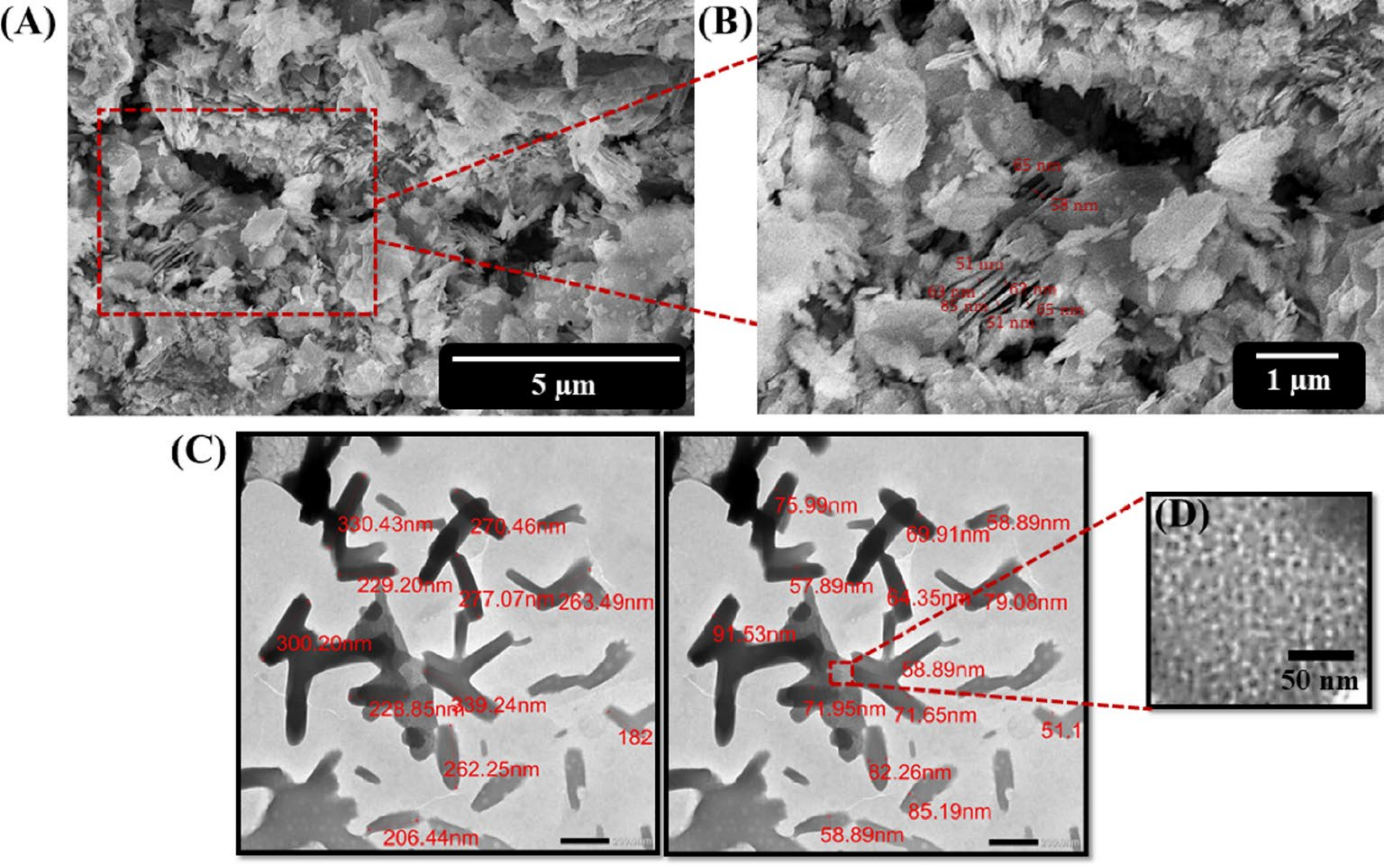
SEM micrographs of Bi-OF nanosheets at **(A)** low, and **(B)** high magnifications. TEM micrographs of Bi-OF nanosheets at **(C)** low, and **(D)** high magnifications.

SEM images were obtained to investigate the morphology of Bi-OF powder. As demonstrated in (Fig. 2A, B), the resulting product clearly displayed a nanosheets-like shape morphology with average nanosheets thickness of 62.5 nm. To further elucidate the topographical characteristics of the framework, TEM images were obtained to investigate the Bi-OF powder, as illustrated in (Fig. 2C, D). The TEM image of the Bi-OF powder, shown in (Fig. 2C), displays nanosheets with an average length to diameter of 262.69 nm, and 71.75 nm, which are consistent with the findings from SEM images presented in (Fig. 2A, B). Notably, at higher magnification (Fig. 2D), the TEM image of the Bi-OF nanosheets distinctly reveals the presence of structural pores.

**Fig. 3:**
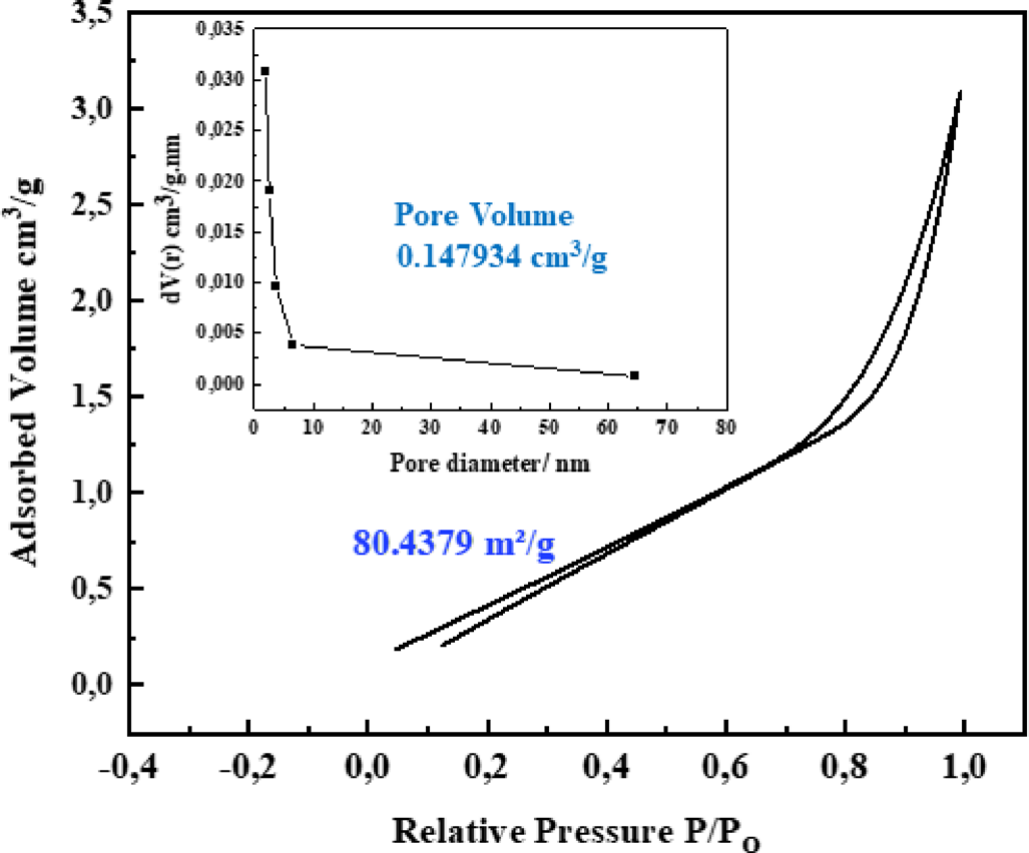
N_2_ adsorption-desorption isotherms, and Dollimore-Heal (DH) method for pore size distribution of Bi-OF nanosheets (inset).

The N_2_ adsorption-desorption isotherm curve for Bi-OF, displayed in (Fig. 3), illustrates the mesoporous nature of Bi-OF. The isotherm exhibits an H_3_ hysteresis loop within the P/P₀ range of 0.8–0.99, indicative of the formation of aggregates of plate-like particles that create slit-shaped pores^24^. This hysteresis loop suggests that the surface possesses a degree of mesoporosity^37^. The BET specific surface area, derived from the N₂ adsorption isotherm, is calculated to be 80.44 m^2^. g^-1^. The mesoporous nature of Bi-OF contributes to reduced resistance pathways, thereby enhancing the electron and ion transport performance of Bi-OF-based supercapacitors. The Barrette–Joyner–Halenda pore size distribution is illustrated in (Fig. 3; inset), the distribution in pore diameter of Bi-OF was mainly in the range from 1.9 nm to 7 nm, with a total pore volume of 0.147934 cm.g^-1^. This distribution strongly indicates the presence of numerous mesoporous structures within Bi-OF. It is well established that a rich mesoporous configuration enhances the longevity and rate capabilities of supercapacitors. This is because mesoporous structures possess a high specific surface area, which facilitates efficient charge transfer, reduces ion diffusion path lengths, and provides sufficient space to accommodate volume changes during charge-discharge cycles.

### Electrochemical measurements

**Fig. 4:**
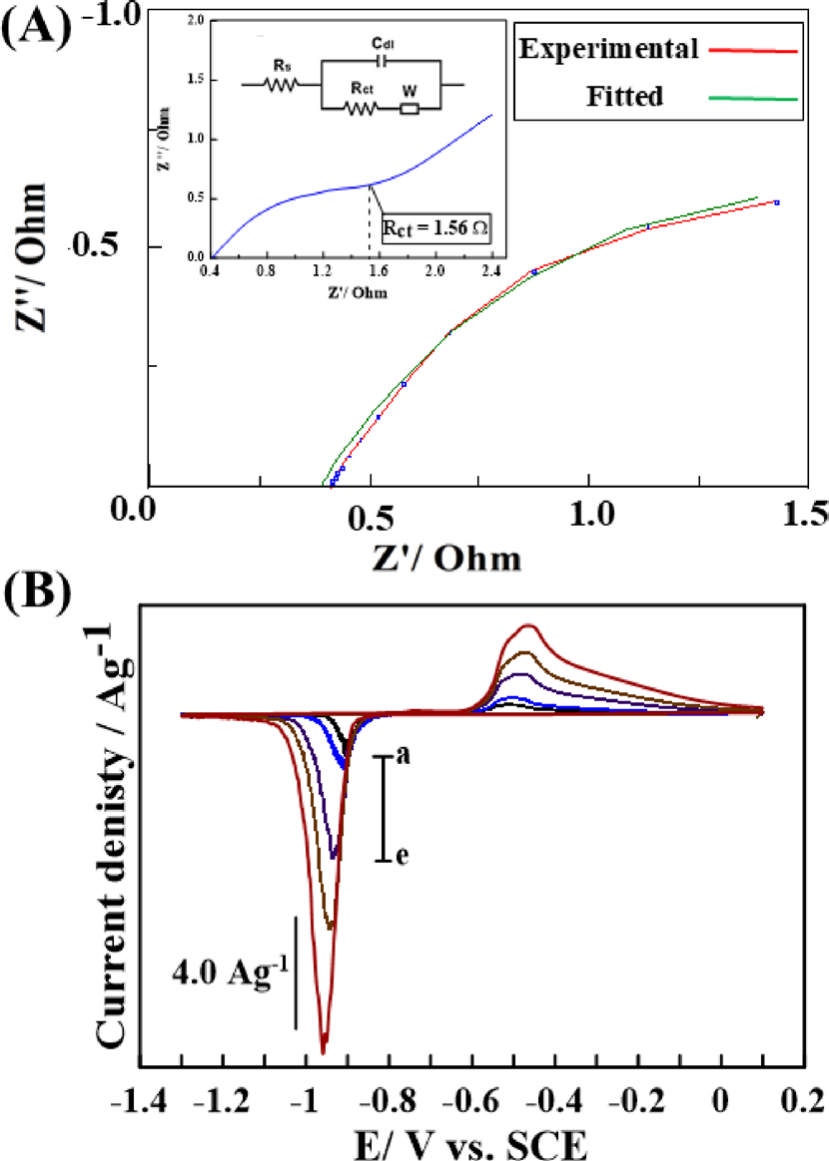
(**A**) The Nyquist plot of Bi-OF electrode. (**B**) CV voltammograms of Bi-OF electrode at (a) 5, (b) 10, (c) 30, (d) 50, and (e) 100 (*v* mV. s^−1^).

The electrochemical properties of as-prepared Bi-OF GCE were investigated via EIS, CV, and GCD measurements, which were carried out to explore the specific capacitance, degree of resistance, and outstanding stability of fabricated electrode. Significantly, the morphology of Bi-OF, characterized by its ultrathin nanosheets using SEM analysis as shown in **(**Fig. 2A), provides abundant electroactive sites conducive to the Faradaic redox reaction, as well as a direct pathway for electron transfer and electrolyte ion diffusion^12^. The Nyquist plot of Bi-OF GCE is illustrated in (Fig. 4A), covering a frequency range from 10^6^ to 0.01 H_z_ in a 1.0 M KOH electrolyte, with the fitting circuit shown in the inset. The plot features a prominent semicircle in the high-frequency region and a straight line in the low-frequency domain^38^.

The solution resistance (R_s_), corresponding to the high-frequency intercept on the real axis of the Nyquist plot, was determined to be 0.39 Ω. This relatively low value indicates good ionic conductivity within the electrolyte, as evaluated using (the ZView software). The diameter of the semicircle, which represents the charge transfer resistance (R_ct_), was measured to be 1.56 Ω, reflecting the resistance associated with redox reactions at the electrode-electrolyte interface^39^. Additionally, the value of R_p_ was found to be 2.26 Ω, while R_ct_ remained at 1.56 Ω, suggesting that R_ct_ is a distinct component in the equivalent circuit. This supports a model of the form R_s_ – [(CPE || R_ct_) + R_p_].In this configuration, R_p_ may represent resistance from a passive surface layer or diffusion-related processes, while R_ct_ specifically corresponds to charge transfer kinetics. Ionic resistance is influenced by both the resistance of the electrolyte within the pores and the diffusion-related resistance of ions navigating through narrow pores^40^.

To explore the supercapacitive behavior, CV measurements of Bi-OF GCE, as demonstrated in (Fig.4B) were recorded in 10.0 mL of 1.0 M KOH at different scan rate (*v*) of 5, 10, 30, 50, and 100 mV.s^−1^ accompanied using Hg/ Hg_2_Cl_2_ and platinum electrodes as reference and counter electrodes, respectively. The cyclic voltammograms clearly displayed redox peaks related to the transition of Bi^+3^/Bi⁰. The redox peak potentials of Bi-OF resemble those of the Bi_2_O_3_^41^, and Sr_0.25_Bi_0.75_O_1.36_^42^ in the presence of OH^−^ ions of the supporting electrolyte. The peaks observed at approximately − 0.5 V during the anodic sweep can be attributed to the oxidation of Bi^0^ to Bi^+3^ as follows: Bi°_(s)_ + 3OH^−^_(aq)_ → Bi(OH)_3(s)_ + 3e^-^. While the peak at approximately − 0.9 V during the cathodic sweep is associated with the reduction of Bi^+3^ to Bi^0^ as follows: Bi(OH)_3__(s)_ + 3e^-^. → Bi°_(s)_ + 3OH^−^_(aq)_. The observation that the cathodic peak is higher than the anodic peak may be attributed to the Bi(OH)_3_ formed from the anodic sweep and Bi(OH)_3_ formed from the interaction of hydroxide ions with the carboxylate groups in the Bi-OF, which contains BDC linkers. Hydroxide ions can deprotonate carboxyl groups, converting them into carboxylate anions and water:

$$\text{Bi-OF-R-COOH+OH}^-\rightarrow\text{Bi-OF-R-COO}^-+\text{H}_2\text{O}$$

In a strongly alkaline medium (1.0 M KOH), the deprotonated Bi-OF may further react to form bismuth hydroxide (Bi(OH)₃) as follows:

$$\text{Bi-OF-R-COO}^-\text{+3OH}^-\rightarrow\text{Bi-OF-R-COO}^-+\text{Bi}\text{(OH)}_3$$

According to the literature^38^, this transformation into Bi(OH)₃ results in a significant reduction in lattice volume and an increase in specific surface area due to the “crystal-crystal conversion” mechanism. This process causes substantial particle contraction, formation of new micropores, and enlargement of initial pores, leading to an increased average pore diameter, total pore volume, and enhanced specific surface area.

Furthermore, the peak current intensity progressively increases as the scan rates rise to 100 mV. s^−1^. Consequently, the CV curves exhibit a distinct redox peak, with one anodic peak (at positive current density) and one cathodic peak (at negative current density) in each curve. These peaks correspond to the relatively sluggish kinetics of the electrochemical reactions at higher scan speeds, implying that the electron transfer mechanism is reversible or quasi-reversible. The higher reduction peak intensity observed for Bi-OF can be explained by the strong electron-accepting nature of Bi³⁺ centers, which favor efficient reduction during the cathodic scan. The porous structure of the Bi-framework enhances ion transport and access to active sites, supporting higher current response. In contrast, the oxidation process may involve slower kinetics or partial structural changes, leading to lower peak intensity^43^.

The specific supercapacitance values (SCs, measured in F. g^−1^) at scan rates of 5, 10, 30, 50, and 100 mV s^−1^ obtained from the CV measurements using Eq. (1) are determined as follows: 1963.78 C. g^−1^ (1402.7 F. g^−1^), 1722.84 C. g^−1^ (1230.6 F. g^−1^), 1566.32 C. g^−1^ (1118.8 F. g^−1^), 1445.78 C. g^−1^ (1032.7 F. g^−1^), and 1154.58 C. g^−1^ (824.7 F. g^−1^), respectively. Moreover, the positions of oxidation and reduction peaks undergo a slight shift towards more positive and negative potentials as the scan rate increases, primarily due to electrode polarization^18,44^.

**Fig. 5:**
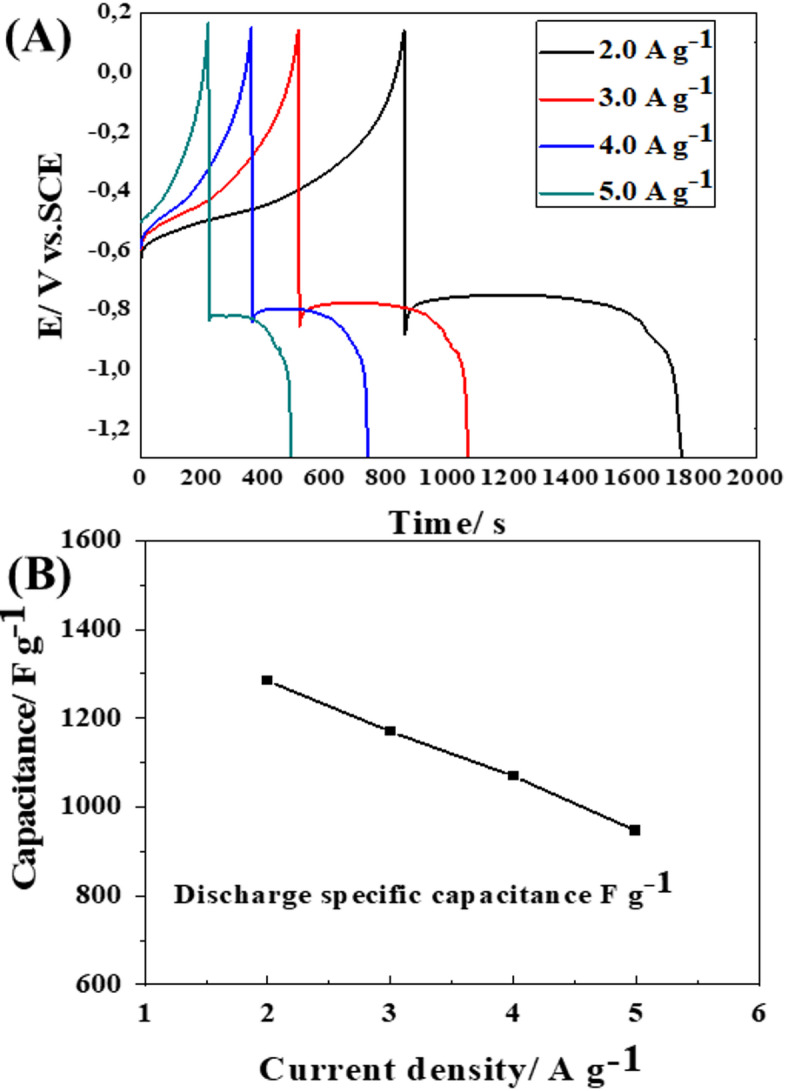
(**A**) GCD curves of Bi-OF electrode different a current density (A. g ^−1^), and (**B**) corresponding plot.

Moreover, the galvanostatic charge-discharge (GCD) profiles of Bi-OF electrode were obtained at varying current densities (2.0, 3.0, 4.0, and 5.0 A. g^−1^), aiming to assess their specific capacitances, as depicted in (Fig. 5A). The specific capacitance of the Bi-OF electrode at 2.0, 3.0, 4.0, and 5.0 A. g^−1^ estimated to be 1797.88 (1284.2 F. g^−1^), 1638.20 (1170.14 F. g^−1^), 1497.58 (1069.7 F. g^−1^), and 1325.52 C. g^−1^ (946.8 F. g^−1^), respectively. Notably, the specific capacitance of the Bi-OF electrode at 2.0 A. g^−1^ is 1797.88 C. g^−1^ (1284.2 F. g^−1^), and it retains a high value of 1325.52 C. g^−1^ (946.8 F. g^−1^) at 5.0 A. g^−1^. As depicted in (Fig. 5B), the proper rate capability observed in the GCD measurements, with capacitance retention of 73.7% at 5.0 A. g^−1^, agrees with the findings from the EIS analysis, which exhibited low R_ct_ and efficient ion diffusion (small W_0_ value). These results indicate that the Bi-OF GCE electrode has both fast electron transfer and effective ion diffusion, which contributes to its high performance at varying current densities^45^.

Since the scientific approach always seeks to compare newly reported results of supercapacitors with commercial supercapacitors. As displayed in (Table 1), Maxwell’s BCAP3000 exhibits the highest specific capacitance at 3000 F. g^−1^^46^, whereas the Bi-OF achieves 1284.2 F. g^−1^, suggesting greater energy storage potential in commercial options, albeit for different applications. The SkelCap 300 stands out with a current density of 5 A. g^−1^, facilitating rapid charge/discharge, compared to the 2.0 A. g^−1^ of Bi-OF. Moreover, the commercialization of graphene-based supercapacitors (SCs) is exemplified by Skeleton Technologies and Ningbo CRRC New Energy Technology^47^. In terms of cycling stability, Bi-OF retains 84.7% capacity after 3000 cycles, comparable to or better than many commercial supercapacitors designed for durability. Thus, while Maxwell and Skeleton models excel in specific capacitance and current density, Bi-OF nanosheets present promising characteristics for targeted applications in sustainable energy storage, highlighting the strengths of each in diverse energy storage contexts.

**Fig. 6:**
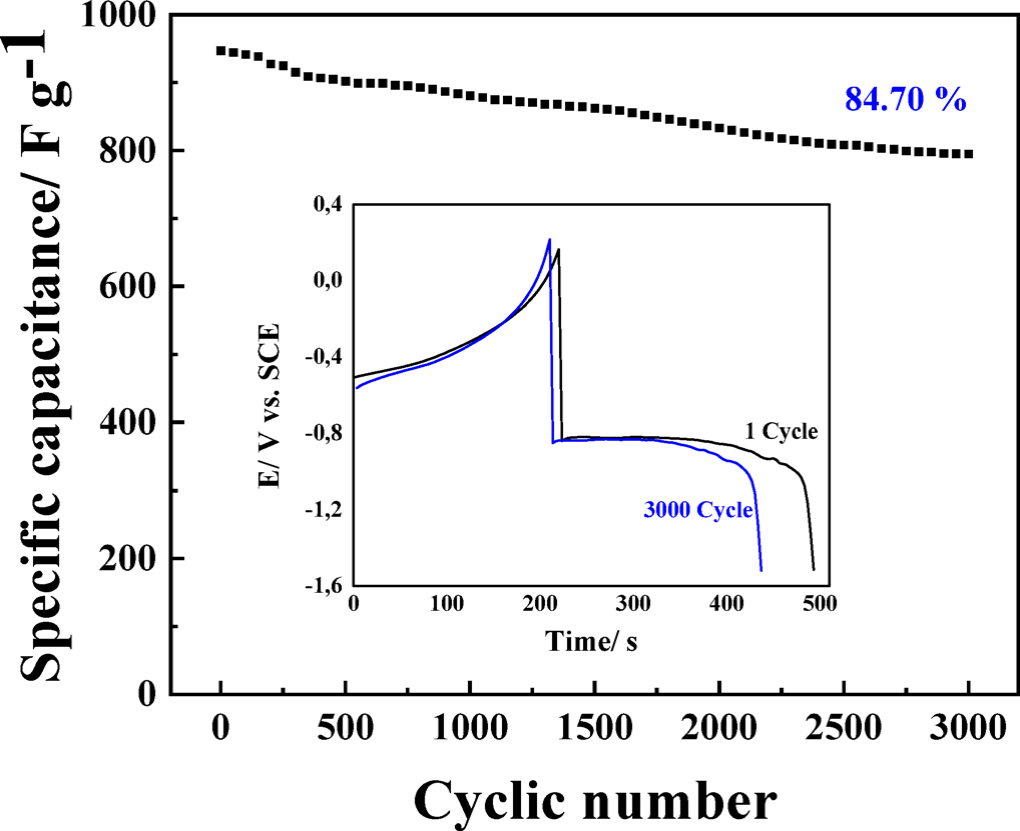
The cycling stability at 5 A. g^−1^ over 3000 cycles.

The cycling stability of the Bi-OF electrode was tested by GCD at 5.0 A. g^−1^. As shown in plot (Fig. 6), Bi-OF electrode exhibited a gradual decrease in specific capacitance, reaching 801.9 F. g^−1^ over 3000 consecutive cycles, and the capacitance retention is 84.7% of the initial capacitance (946.8 F. g^−1^), denoting the outstanding cycling stability. During cycling, the formation and breakdown of Bi(OH)₃ in the Bi-OF causes changes in its structure^38^. Repeated reactions between Bi and Bi(OH)₃ create mechanical stress, leading to degradation. At first, these changes, caused by a “crystal-crystal conversion” mechanism, reduce the lattice volume and increase the specific surface area, which improves pore characteristics^38^. However, over time, this process leads to particle contraction, the creation of new micropores, and the enlargement of existing pores. Ultimately, these effects weaken the structure and reduce the cycling stability of the Bi-OF.

Upon comparing the Bi-OF electrode with other Bi-based nanocomposites listed in (Table 2), we noted that the Bi-OF synthesized in a simple method displayed remarkable electrochemical properties compared to previously reported nanocomposites, demonstrating acceptable stabilityperformance. Considering the characteristics of the fabricated electrodes discussed previously, it can be concluded that the Bi-OF electrode showed a desirable level of capacitance with low resistivity and maintained suitable stability even after 3000 cycles, making it a promising candidate for application in supercapacitors.

**Table 1:** Electrochemical capacitance performance of Bi-based nanosheets electrode with recent commercial supercapacitors.

Parameter	Commercial supercapacitors	Bi-OF electrode
Specific Capacitance (F. g^−1^)	3000 (Maxwell)^46^	1284.2
Current Density (A. g^−1^)	0.6 (Maxwell)^46^, 5 (Skeleton)^47^, 1 (Ningbo)^47^	2.0
Cycling Stability	Varies, often ≥ 80% after 1000 cycles	84.7% after 3000 cycles

**Table 2:** Electrochemical capacitance performance of different types of Bi-based nanosheets electrode.

Electrode	Capacitance F. g^−1^	Current density A. g^−1^	Retention rate %/Cycles	Refs.
Bi_2_O_3_@C	1378	0.5	93/4000	^30^
Bi-OF-derived Bi_2_O_3_/C hollow nanofibers	1420	1.0	92.21/4000	^48^
Bi_2_Se_3_@C	565.9	1.0	90.5/1000	^31^
Bi-Bi_2_O_3_/CNT core-shell	850	1.0	72.9/1000	^28^
3D activated carbon fiber paper/α-Bi_2_O_3_	906	1.0	73.56/5500	^29^
Bi_2_S_3_/sulfur-doped g-C_3_N_4_	670	1.0	80/3000	^32^
Bi^3+^/H_3_BTC	896.1	0.5	79.5/1000	^48^
**Bi-OF**	**1284.2 (1797.88 C. g** ^**–1**^ **)**	**2.0**	**84.7/3000**	**This study**

## Conclusion

This study presents a calcination-free solvothermal strategy for synthesizing Bi-OF nanosheets with a Bi^3+^/H_2_BDC molar ratio of 1:1, aimed at overcoming the limitations of conventional battery-type electrode materials such as low conductivity and poor cycling stability. The proposed method yielded porous Bi-OF nanosheets with preserved structural integrity and accessible redox-active sites, eliminating the need for high-temperature treatment that often degrades frameworks. The Bi-OF modified electrode exhibited superior electrochemical performance, achieving a high specific capacitance of 1797.88 C. g^−1^ (1284.2 F. g^−1^) at 2.0 A. g^−1^, low internal resistance (1.56 Ω), and excellent cycling stability with 84.7% retention after 3000 cycles at 5.0 A. g^−1^. These results confirm that the calcination-free approach enhances ion transport and redox site utilization, offering a sustainable and efficient alternative to conventional MOF-derived electrodes. The findings position Bi-OF as a promising candidate for next-generation supercapacitor applications, combining high energy storage capacity with environmental and economic advantages.

## Data Availability

All data generated and/or analyzed during the current study are included in this published article.
